# Impact of catheterization on shear-mediated arterial dilation in healthy young men

**DOI:** 10.1007/s00421-020-04473-8

**Published:** 2020-08-28

**Authors:** Andrea Tryfonos, Matthew Cocks, Debar Rasoul, Joseph Mills, Daniel J. Green, Ellen A. Dawson

**Affiliations:** 1 Research Institute for Sport and Exercise Science, Liverpool John Moores University, Liverpool, L3 3AF UK; 2 Liverpool Heart and Chest Hospital, Liverpool, L14 3PE UK; 3 School of Human Sciences (Exercise and Sport Science), The University of Western Australia, Crawley, WA 6009 Australia

**Keywords:** Catheterization-induced damage, Flow-mediated dilation, Healthy young males, Intact endothelium

## Abstract

**Purpose:**

Animal studies have shown that endothelial denudation abolishes vasodilation in response to increased shear stress. Interestingly, shear-mediated dilation has been reported to be reduced, but not abolished, in coronary artery disease (CAD) patients following catheterization. However, it is not known whether this resulted from a priori endothelial dysfunction in this diseased population. In this study, we evaluated shear-mediated dilation following catheterization in healthy young men.

**Methods:**

Twenty-six (age: 24.4 ± 3.8 years, BMI: 24.3 ± 2.8 kg m^−2^, *V*O_2peak_: 50.5 ± 8.8 ml/kg/min) healthy males underwent unilateral transradial catheterization. Shear-mediated dilation of both radial arteries was measured using flow-mediated dilation (FMD) pre-, and 7 days post-catheterization.

**Results:**

FMD was reduced in the catheterized arm [9.3 ± 4.1% to 4.3 ± 4.1% (*P* < 0.001)] post-catheterization, whereas no change was observed in the control arm [8.4 ± 3.8% to 7.3 ± 3.8% (*P* = 0.168)]. FMD was completely abolished in the catheterized arm in five participants. Baseline diameter (*P* = 0.001) and peak diameter during FMD (*P* = 0.035) were increased in the catheterized arm 7 days post-catheterization (baseline: 2.3 ± 0.3 to 2.6 ± 0.2 mm, *P* < 0.001, peak: 2.5 ± 0.3 to 2.7 ± 0.3 mm, *P* = 0.001), with no change in the control arm (baseline: 2.3 ± 0.3 to 2.3 ± 0.3 mm, *P* = 0.288, peak: 2.5 ± 0.3 to 2.5 ± 0.3 mm, *P* = 0.608).

**Conclusion:**

This is the first study in young healthy individuals with intact a priori endothelial function to provide evidence of impaired shear-mediated dilation following catheterization. When combined with earlier studies in CAD patients, our data suggest the catheterization impairs artery function in humans.

## Introduction

The endothelium plays a fundamental role in the regulation of vascular tone (Sandoo et al. 2010; Green et al. 2017). Previous studies performed in animals have demonstrated that, following endothelial denudation, arteries no longer respond to increased blood flow (Pohl et al. 1986) or exercise (Berdeaux et al. 1994), reflecting the critical role of endothelial cells in the regulation of vascular tone in vivo. However, Dawson et al. (2010a, b) showed depressed, but not abolished, radial artery shear-mediated dilation following transradial catheterization in CAD patients (Dawson et al. 2010a). This finding combined with studies showing that flow-mediated dilation (FMD) was not completely abolished by endothelial nitric oxide synthase (eNOS) inhibition (Stoner et al. 2012; Pyke et al. 2010; Green et al. 2014, 2011; Wray et al. 2013), or nitric oxide (NO) and prostaglandin (PGs) double blockade (Thijssen et al. 2011; Schrage et al. 2004), raises the question of whether other endothelium-dependent or -independent mechanisms may contribute to the FMD response. To the best of our knowledge, no previous study has assessed FMD responses following endothelial damage/denudation in healthy humans.

In the current study, we aimed to assess radial artery shear-mediated dilation following catheterization in young trained males, in whom the endothelial layer is assumed to be healthy and functional. FMD was used to assess shear-mediated arterial responses in both radial arteries before, and 7 days post-, catheterization. This time frame is clinically relevant, as it is common to begin exercise-based cardiac rehabilitation around 1 week post-catheterization. We hypothesized that catheterization would impair endothelium-mediated arterial dilation in response to a shear stress stimulus in these healthy men.

## Methods

### Participants

Thirty-one healthy young (< 35 years), trained males (≥ 150 min of moderate-intensity or ≥ 75 min of high-intensity exercise per week) with a BMI < 32 were recruited. Participants were non-smokers and were free of cardiovascular disease (CVD), or CVD risk factors such as diabetes, hypertension or hypercholesterolemia. None reported taking medications or any drugs that would impact vascular function. Informed consent was gained from all participants prior to the experimental procedures. The study conformed to the Declaration of Helsinki, and ethical approval was obtained from the Liverpool East NHS Research Ethics Committee (18/NW/0428).

### Study design

Participants attended the cardiovascular laboratory at Liverpool John Moores University on three occasions: (a) baseline, (b) catheterization and (c) follow-up. All experimental procedures were conducted between 7 am and 1 pm, in a quiet temperature-controlled room, and participants fasted overnight and instructed to abstain from caffeine (> 8 h), alcohol and vigorous exercise (> 24 h) before each visit, in accordance with current guidelines (Thijssen et al. 2019). During the baseline visit, radial artery (RA) shear-mediated dilation was assessed in both arms using FMD. Following FMD, peak oxygen consumption (*V*O_2peak_) during maximal graded exercise on a cycle ergometer (Lode Excalibur Sport Cycle Ergometer, The Netherlands) was also assessed, using a gas analysis system (MOXUS Metabolic Cart (AEI Technology, USA) (Cocks and Wagenmakers 2016; Medbø et al. 2012; Beltrami et al. 2014). Briefly, participants started cycling at 60 W for 3 min; following this, the workload was increased by 35 W every 3 min until volitional fatigue. *V*O_2peak_ corresponds to the highest value achieved over a 15 s recording period. At least 48 h following baseline, participants attended the catheterization trial, where a transradial catheter was inserted into the participants’ RA (CATH arm). Seven days following catheterization participants attended the laboratory for the follow-up visit, where FMD was again assessed in the catheterized arm, while the contralateral arm was used as an internal control (CON arm).

### Transradial catheterization

An 18–20-gauge catheter (0.9–1.2 mm diameter, 8–10 cm length) (leadercath, Vygon, UK) was inserted into the right RA (15.4% via left RA), under local anaesthesia (2-4 ml Marcain Polyamp steripack 0.5%, Aspen), by a cardiologist. Shortly after catheterization, two (5 participants) or four (21 participants) separate flexible J-shaped guide wires (paediatric J-wires, 0.46 mm, 40 cm length, Vygon, UK) were advanced 3–4 cm beyond the tip of the catheter and run back and forth to collect endothelial cells from the inside of the RA, before being removed (Colombo et al. 2002; Feng et al. 1999). Catheters were removed at least 1 h after insertion (1–4 h), and manual pressure was placed on the catheterized area for approximately 10 min to stop the bleeding.

### Bilateral radial FMD

Following at least 10 min of supine rest, blood pressure and heart rate were measured using an automated sphygmomanometer (GE Pro 300V2, Dinamap, Tampa, FL, USA). RA shear-mediated dilation was then measured in both arms using FMD, as described previously (Dawson et al. 2010a). Briefly, an optimal B-mode image of the RA was acquired, using a 12-MHz multi-frequency linear array probe, attached to a high-resolution ultrasound machine (T3000; Terason, Burlington, MA) to image the RA 10–15 cm proximal to the wrist. This section was above of the catheter. Relative diameter change, time to peak (following cuff release) and shear rate area under the curve (SRAUC) were analysed by the same blinded observer, using custom-designed edge-detection and wall-tracking software (Woodman et al. 2001; Thijssen et al. 2011, 2019). FMD was reported as the maximum percentage change in artery diameter from baseline to peak when the cuff was released, as described in detail previously (Thijssen et al. 2011, 2019). The same ultrasounds and sonographers were used between visits, and within participants.

### Statistical analysis

All analyses were performed using IBM SPSS statistics for Windows, version 25.0. Armonk, NY: IBM Corp. Allometric scaling was performed to control for differences in baseline diameter (Atkinson and Batterham 2013), and a linear mixed model with covariate control for scaled baseline diameter and SRAUC was used to determine the main effect of time and arm. A linear mixed model was also used to analyse the differences in baseline diameter, peak diameter, time to peak, SRAUC, and mean blood flow area under the curve (AUC) during FMD. Pairwise comparisons were performed when significant main or interaction effects were detected, using Fisher’s least significant difference (LSD) test. Pearson correlation analysis was used to determine relations of interest. Paired *t* tests were used to assess differences in haemodynamic parameters, pre- and post-catheterization. Results are presented as mean ± SD, and significance was set at *P* ≤ 0.05.

## Results

Twenty-six participants (age: 24.4 ± 3.8 years, body mass index (BMI): 24.3 ± 2.8 kg m^−2^) completed all three experimental visits. All participants completed at least ≥ 150 min of moderate-intensity or ≥ 75 min of high-intensity exercise per week. Mean peak oxygen consumption (*V*O_2peak_) was 50.5 ± 8.8 ml/kg/min. Out of 104 FMD scans (2 arms, pre- and post-catheterization, 26 participants), 3 scans were excluded from further analysis (2 temporary radial artery spasm in the catheterized artery, 1 poor-quality scan).

### Effects of catheterization on radial FMD

**Fig. 1:**
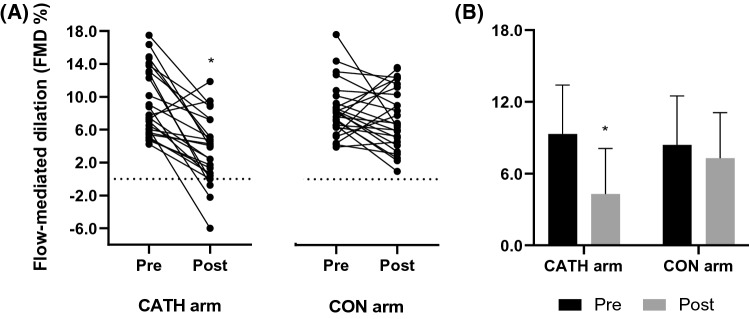
Flow-mediated dilation (FMD %) in the catheterized (CATH) and contralateral (CON) radial arteries, pre- and 7 days post-catheterization (Post). Individual responses (**a**) and summary data, presented as mean ± SD, *n* = 26 (**b**). A mixed linear model (arm × time) with Fisher’s least significant difference post hoc for pairwise comparisons was used. *Significantly different from the catheterized arm pre (*P* < 0.05)

There was no significant difference in FMD between arms, pre-catheterization (*P* = 0.303). There was a significant interaction (time × arm) for FMD, after controlling for baseline diameter (*P* = 0.004) (Fig. 1), SRAUC (*P* = 0.003) and both covariates (*P* = 0.01). However, baseline diameter and SRAUC were not significant covariates in this model, *P* = 0.064 and *P* = 0.122, respectively. There was also a main effect for time (*P* = 0.02), arm (*P* < 0.0001) and intervention (time × arm, *P* = 0.001), when peak FMD% was assessed without allometric scaling. Post hoc assessment revealed that there was a significant reduction in FMD in the catheterized arm pre- to 7 days post-catheterization (9.3 ± 4.1% to 4.3 ± 4.1%; *P* < 0.001). FMD was completely abolished in 5, and was < 1% in a further 2, of the 26 participants. In contrast, there was no significant change in FMD in the control arm (8.4 ± 3.8% to 7.3 ± 3.8%; *P* = 0.168). Finally, SRAUC (time × arm *P* = 0.189), time to peak (time × arm *P* = 0.664) and mean blood flow AUC (time × arm *P* = 0.949) were not different between arms or pre- to post-catheterization (Table 1).

**Table 1:** Baseline dimeter, peak diameter, time to peak, shear rate area under the curve (SRAUC) and mean blood flow area under the curve (AUC) before (pre) and at 7 days post-catheterization (post), in the catheterized (CATH) arm and the contralateral (CON) arm

	CATH arm		CON arm	
	Pre	Post	Pre	Post
Baseline diameter (mm)	2.32 ± 0.28	2.62 ± 0.28^*^	2.26 ± 0.23	2.32 ± 0.25^†^
Peak diameter (mm)	2.54 ± 0.32	2.72 ± 0.31^*^	2.46 ± 0.26	2.49 ± 0.28^†^
Time to peak (s)	58 ± 28	66 ± 39	55 ± 25	56 ± 27
SRAUC (s^−1^ 10^3^)	32.9 ± 17.5	27.4 ± 17.8	27.2 ± 14.6	28.7 ± 17.4
Mean AUC flow	88.6 ± 53.4	94.3 ± 62.9	69.9 ± 46.5	76.6 ± 56.1

Results are presented as mean ± SD, *n* = 26. A mixed-linear model (arm × time) with Fisher’s least significant difference post hoc for pairwise comparisons was used

*Significantly different from pre- catheterization (*P* < 0.05)

^†^Significantly different from CATH arm post-catheterization (*P* < 0.05)

**Fig. 2:**
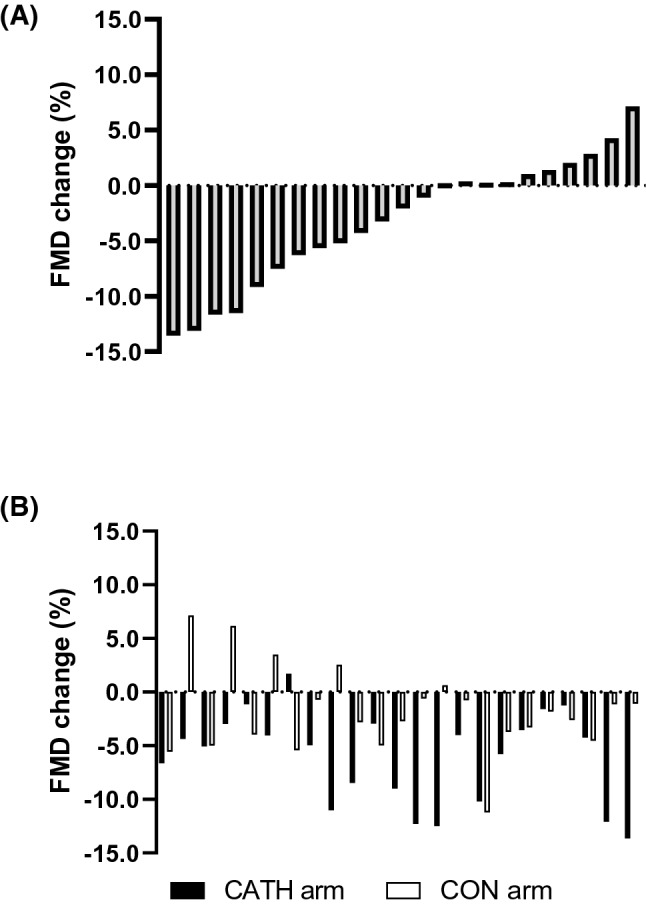
**a** Flow-mediated dilation (FMD %) change in the catheterized arm, pre- to post-catheterization, after accounting for change in the control arm, pre- vs post-catheterization. **b** Individual FMD % change of each catheterized (CATH) and control (CON) arm (*n* = 23)

To isolate the magnitude of local FMD change as a result of catheterization, we calculated the FMD change in the catheterized arm (pre- to post-catheterization), then subtracted the change in FMD in the control arm (pre- to post-catheterization) (Fig. 2a). Figure 2b indicates FMD% change in each arm, in all individuals. In keeping with the data above, there was a significant reduction in FMD (> 5%) as a result of catheterization in 9 out of 23 participants. This FMD change was not correlated with the baseline diameter in the catheterized artery prior to catheterization (*r* = 0.300, *P* = 0.164), or with participants’ age (*r* = 0.217, *P* = 0.320), or BMI (*r* = − 0.244, *P* = 0.263), or *V*O_2peak_ (*r* = 0.344, *P* = 0.108). Catheters with external diameter of 0.9 mm and 1.2 mm were used in 16 and 7 participants, respectively. There was no impact of catheter size on FMD change following catheterization (*r* = − 0.179, *P* = 0.413). Artery-to-sheath ratio (arterial diameter/external diameter of the catheter) was not associated with FMD change following catheterization (*r* = 0.291, *P* = 0.177). In addition, FMD change was not associated with the number or J wires used (2 or 4) (*r* = − 0.316, *P* = 0.142) or the duration of catheterization (*r* = 0.104, *P* = 0.634).

### Effects of catheterization on arterial diameter

There was a significant interaction effect (time × arm) for baseline diameter (*P* = 0.001) (Table 1), demonstrating an increase in baseline diameter in the catheterized arm 7 days post-catheterization, when compared with pre-catheterization (*P* < 0.001) or when compared to the contralateral control arm 7 days post-catheterization (*P* < 0.001). There was no change in the control arm pre- to 7 days post-catheterization (*P* = 0.288). In addition, a significant interaction effect (time × arm) was shown for peak diameter, as assessed during FMD (*P* = 0.035) (Table 1). When pairwise comparisons were performed, peak diameter in the catheterized arm was higher 7 days following catheterization compared to pre-catheterization (*P* = 0.001) or in the contralateral control arm (*P* < 0.001), whereas no change in peak diameter was reported in the control arm pre- to post-catheterization (*P* = 0.608).

### Haemodynamic measurements

There was no change in systolic blood pressure, diastolic blood pressure, mean arterial pressure or heart rate from baseline to follow-up (*P* > 0.05). Data are reported in Table 2.

**Table 2:** Resting haemodynamic measurements pre-catheterization (pre) and 7 days post-catheterization (post)

	Pre	Post	*P* value
SBP (mmHg)	116 ± 9	113 ± 7	0.220
DBP (mmHg)	63 ± 7	60 ± 6	0.161
MAP (mmHg)	79 ± 13	77 ± 12	0.089
HR (beats per min)	61 ± 6	62 ± 5	0.435

Results are presented as mean ± SD, *n* = 26

*SBP* systolic blood pressure, *DBP* diastolic blood pressure, *MAP* mean blood pressure, *HR* heart rate

## Discussion

The aim of this study was to determine the impact of radial artery catheterization on shear-mediated dilation in healthy, young trained males with preserved a priori endothelial function. We demonstrated that catheterization increased baseline diameter and impaired shear-mediated dilation in the catheterized RA. Indeed, shear-mediated dilation was abolished in almost one-fifth of participants following catheterization. Neither baseline diameter, age, BMI, fitness level (*V*O_2peak_), catheter size, artery-to-sheath ratio, duration of catheterization or number of J wires predicted FMD impairment in the catheterized RA.

Although the critical role of the endothelium in the regulation of vascular tone has been studied previously, such information is from either animal models (Berdeaux et al. 1994; Pohl et al. 1986) or patients undergoing transradial catheterization for coronary angiography and/or angioplasty (Mitchell et al. 2017; Burstein et al. 2007; Dawson et al. 2010a, b; Tryfonos et al. 2020). In the latter case, endothelial dysfunction is likely to be apparent prior to catheterization (Neunteufl et al. 1997). To our knowledge, this is the first study providing direct evidence of impaired shear-mediated dilation following catheterization in young healthy trained individuals with an optimally functioning endothelial layer a priori. Interestingly, we observed completely abolished FMD in one-fifth of our healthy participants following catheterization. The magnitude of impairment (complete abolition vs partial impairment) may be due to several factors. The external diameter of the catheter sheaths used in this study were 0.9 or 1.2 mm and, although mean pre-procedure internal radial artery diameter was ~ 2.3 mm, this ranged from 1.7 to 2.9 mm. J-shaped guidewires used to harvest endothelial cells were the same in all participants, but some subjects had four J wires (*n* = 21) and others two J wires (*n* = 5). Despite the fact that these factors did not impact the FMD results statistically, we cannot entirely rule out the possibility of some impact.

Our data from young trained individuals essentially support animal studies of endothelial denudation, in which balloon inflation or catheterization abolished FMD (Pohl et al. 1986; Berdeaux et al. 1994). We previously reported reduced FMD in CAD patients, with a similar degree of FMD impairment, *on average*, as that observed in the current study. Nevertheless, none of our previous CAD patients exhibited complete abolition of the FMD response 24 h post catheterization, despite the use of larger catheters (*n* = 13) in those subjects (Dawson et al. 2010a). Recently published data from our group further support the notion that FMD is impaired, but not abolished, in CAD patients (*n* = 33) 7 days post-denudation (Tryfonos et al. 2020). In the current study, J-shaped wires (external diameter 3 mm) were used in addition to catheters (Tryfonos et al. 2020), which may explain some of the differences between studies. It is also possible that healthy subjects and those with CAD possess endothelial cells that are differentially regulated. Indeed, we previously speculated that the presence of dilation following catheterization may suggest that a presence of a shear-mediated but endothelium-independent mechanism (Dawson et al. 2010a). In support of this hypothesis, a review by Green et al. (2014) suggested that the contribution of nitric oxide (NO) to FMD may be smaller in CVD patients, compared to healthy individuals (Green et al. 2014). Oxidative stress and inflammation increase post-endothelial denudation (Berg et al. 2004) and may remain elevated for 1–15 days (Gong et al. 1996; Szocs et al. 2002; Nunes et al. 1997; Azevedo et al. 2000). Such pathways may also contribute to differential impacts on post-catheterization responsiveness in healthy versus diseased arteries. We did not assess specific molecular pathways (or smooth muscle function) in the present study, but future experiments could focus on individual pathways within the vessel wall to elucidate the mechanisms of shear-mediated dilation.

Baseline diameter was higher in the catheterized artery 7 days post-catheterization compared to pre-catheterization, while no change was observed in the control artery, supporting the suggestion that catheter impacts were unilateral in nature. Interestingly, we found that peak diameter during FMD, in addition to baseline diameter, was elevated following catheterization. Earlier studies investigating the influence of catheterization in CAD patients have only reported increased baseline diameter, with no effect on peak diameter (Dawson et al. 2010a, 2010b; Mitchell et al. 2017; Burstein et al. 2007; Tryfonos et al. 2020). Consequently, it is unclear whether elevated peak diameter occurs in all populations or is present only in young trained individuals as an advantage of healthier arteries. It is important to reiterate that our findings pertaining to FMD impairment were assessed after allowing for changes in baseline diameter induced by catheterization.

To isolate the local effect of catheterization in the shear-mediated dilation, we calculated the FMD change in the catheterized artery pre–post catheterization and subtracted this from the similarly calculated FMD change in the non-catheterized artery. This approach normalizes for any systematic variability and allowed us to further explore factors that may contribute to FMD impairment following catheterization. Except for six participants whose shear-mediated dilation appeared to not be greatly affected by catheterization, there was a pronounced reduction in FMD response in the catheterized artery following endothelial disruption. Indeed, almost half of the participants (9 out of 23) reported a significant reduction in FMD (> 5%), after normalizing data from the systemic variability. This further supports the localized nature of the impact of catheterization on FMD in young healthy well-trained individuals.

Although smaller RA baseline diameter prior to catheterization has been associated with increased radial artery occlusion risk (Rashid et al. 2016), and a larger magnitude of endothelial dysfunction (i.e. lower radial artery FMD) (Heiss et al. 2009) following PTCA and/or PCI, our data revealed no association between baseline diameter and FMD impairment post-catheterization. In addition, neither catheter size nor artery-to-sheath ratio appeared to result in greater FMD impairment following catheterization. Importantly, in the present study, the baseline arterial diameter (~ 2.3 mm) was larger than the sheath external diameter (0.9–1.2 mm) in all participants, which could explain the absence of correlations between baseline diameter, catheter size and artery-to-sheath ratio with FMD impairment. Indeed, Saito et al. (1999) observed greater flow reduction in the radial artery in patients with artery-to-sheath ratio < 1, compared to those with artery-to-sheath ratio > 1 (Saito et al. 1999). Given the importance of artery-to-sheath ratio to endothelial dysfunction related to catheterization, it could be hypothesized that transfemoral catheterization might result in lesser degrees of arterial damage. However, femoral site catheterization has been associated with impaired brachial FMD (Kitta et al. 2005; Patti et al. 2005), indicating a possible systemic impact on endothelial dysfunction. Transradial catheterization is superior in safety, cost and vascular complications compared to femoral approach (Anjum et al. 2017) and has become more popular in recent years. Although our findings revealed no correlation between FMD impairment and age, BMI, or fitness level, the lack of association in our study may be explained by the relatively small sample size and narrow range of subjects across these variables.

From a clinical perspective, it has been hypothesized that optimizing the function and size of arteries prior to catheterization may limit the impact of transradial catheterization and improve arterial health post-procedure. As such, preoperative exercise-based rehabilitation has been suggested prior to transradial catheterization (Alkarmi et al. 2010), due to the well-established benefits of exercise training on arterial function and outward remodelling (Hambrecht et al. 2004; Green et al. 2017; Dawson et al. 2012; Conraads et al. 2015). Although our current data in fit healthy young subjects indicate that preoperative exercise-based rehabilitation is unlikely to fully negate the impact of catheterization on arterial function, the well-established benefits of exercise training on artery function, structure and health should not be discounted (Green et al. 2017).

This study had a number of limitations. We recruited young healthy males, and given the effect of oestrogen on endothelial function, we should be cautious to generalize our results in females. In addition, we did not control for age, BMI, *V*O_2peak_, catheter size, artery-to-sheath ratio, duration of catheterization and the number of J-shaped wires used. However, we performed Pearson correlations between the FMD change and the aforementioned factors, with no associations revealed. In addition, we did not infuse vasodilators during the catheterization. Given that this is a common practice in transradial catheterization in patients to increase the arterial diameter and eliminate vascular complications, it is likely to affect our outcomes. However, artery-to-sheath ratio was always over 1 and neither artery-to-sheath ratio nor baseline diameter appeared to associate with FMD impairment; therefore, it is unclear whether larger arterial size induced by vasodilators would significantly affect our results, indicating reduced endothelial-dependent dilation 7 days post-catheterization. Finally, we did not evaluate the function of vascular smooth muscle cells (endothelium-independent function) and cannot comment on the relative impact on the endothelium versus vascular smooth muscle.

This study provides novel information indicating that shear-mediated dilation in young healthy trained individuals is impaired, and in one-fifth of participants abolished, as a result of transradial catheterization. Neither baseline diameter, age, BMI, fitness level, nor catheter size, artery-to-sheath ratio, duration of catheterization or number of J wires used were associated with FMD reduction in this experiment. When combined with earlier studies in CAD patients (Dawson et al. 2010a, 2010b; Mitchell et al. 2017; Burstein et al. 2007; Tryfonos et al. 2020), in whom FMD was impaired but not abolished, our data suggests that the impact of catheterization on artery function and FMD may differ between healthy, well-trained individuals and those with a priori endothelial dysfunction. Future studies should examine whether improved endothelial function and greater arterial size (i.e. preoperative cardiac rehabilitation) may decrease endothelial damage following catheterization and address specific mechanisms that are affected by catheterization, including endothelium-dependent and possibly -independent shear-mediated pathways.

## Data Availability

The datasets generated during and/or analysed during the current study are available from the corresponding author on reasonable request.

## Abbreviations

CAD: Coronary artery disease
CATH: Catheterized arm
CON: Control (non-catheterized) arm
CVD: Cardiovascular disease
FMD: Flow-mediated dilation
SRAUC: Shear rate area under the curve
*V*O_2peak_: Peak volume of oxygen consumption
